# Understanding implementation contexts and determinants of e-learning for global health security competency-based training in LMICs

**DOI:** 10.1038/s44401-026-00105-z

**Published:** 2026-07-01

**Authors:** Kehinde O. Ogunyemi, Virgil K. Lokossou, Senait Kebede, Lionel S. Sogbossi, Julian Salim, Ahmed Haji-Said, Morgan Toth, Kelechi Umoga, Adaiah P. Soibi-Harry, Simon Antara, Carol Haley, Affan T. Shaikh, Michael Olaniran, Tolbert Nyenswah, Wondwossen A. Gebreyes, Chima Ohuabunwo, Scott McNabb, Melchior A. Aïssi

**Affiliations:** 1 Hubert Department of Global Health, Rollins School of Public Health, Emory University, Atlanta, GA USA; 2 Department of Epidemiology and Biostatistics, College of Public Health, University of Georgia, Athens, GA USA; 3 West African Health Organization, Abidjan, Côte d’Ivoire; 4 Center for Studies of Human Health, Emory University, Atlanta, GA USA; 5 Department of Global Health, University of Washington, Seattle, WA USA; 6 Public Health Practice LLC, Public Health Practice LLC, Florida, FL USA; 7 Massachusetts General Hospital, Brigham and Women’s Hospital, Harvard Medical School, Boston, MA USA; 8 Department of Infectious Disease, Emory School of Medicine, Emory University, Atlanta, GA USA; 9 African Field Epidemiology Network, Kampala, Uganda; 10 College of Engineering and Computer Science, University of Michigan-Dearborn, Dearborn, MI USA; 11 Department of International Health, Bloomberg School of Public Health, Johns Hopkins University, Baltimore, MD USA; 12 The Global One Health Initiative (GOHi), The Ohio State University, Columbus, OH USA; 13 Department of Community Health and Preventive Medicine, Morehouse School of Medicine, Atlanta, GA USA

**Keywords:** Information systems and information technology, Diseases, Health care, Health humanities

## Abstract

E-learning offers great benefits and potential to improve the capability of the public health workforce to prevent, detect, and respond to public health emergencies. This study aims to identify the implementation contexts and determinants of multi-communication online training (MOT) in low- and middle-income countries (LMICs). Using the updated Consolidated Framework for Implementation Research (CFIR), we mapped previously established MOT constraints and enablers onto its five domains and 39 constructs to determine implementation contexts and determinants, respectively. Overall, contexts for constraints and enablers to MOT implementation were established across all five domains of CFIR, with 16 determinants identified. The contexts were mostly linked to intervention (26.7%), followed by inner setting (23.3%), outer setting (23.3%), process (20.0%), and individuals (6.7%). The top three implementation determinants of MOT identified were intervention *design* (16.7%), outer setting *policies and laws* (13.3%), and outer setting *local conditions* and inner setting *available resources* (10.0%). These findings could be used to guide evidence-informed selection of strategies and prioritization of efforts and resources for e-learning implementation to improve global health security competency-based training in LMICs.

## Introduction

The effectiveness and benefits of e-learning interventions (e.g., improved learning outcomes, reduced costs) are well described in the literature. Yet progress on its widespread utilization for capacity building in many sectors, including public health remains slow in Africa despite advancements in information and communication technology (ICT)^1–5^. More so, as a direct benefit, high-impact e-learning interventions such as a multi-communication online training (MOT) that combines both synchronous and asynchronous online methods with multiple ICT-enabled approaches (e.g., social media-based learning, digital simulation-based learning) have been associated with enhanced disease surveillance, strengthened workplace ICT, and improved emergency preparedness and response (EPR) in Africa and other parts of the world (e.g., Vietnam)^6–13^. This suboptimal integration of e-learning into routine practice (e.g., global health security trainings) in some areas despite its advantages and potentials is, in part, due to weak implementation efforts across design, delivery, and sustainment^14–17^. To close this evidence-to-practice gap, understanding the implementation contexts (i.e., a combination of characteristics that describe how a range of factors relate and interact with an EBI at multiple levels) and implementation determinants (i.e., the characteristics that explain why an EBI works in routine practice) of e-learning could support its successful implementation^17^.

To accelerate the implementation of e-learning interventions in the public health workforce, recent research has explored mostly its constraints and/or enablers^18–21^. But there is a paucity of evidence on the implementation contexts and determinants (ICD) in which these interventions are planned, adopted, and maintained in an African setting^10,22–24^. While evidence of key constraints to e-learning (e.g., unsatisfactory internet quality and costs, unavailability of context-specific trainings, and inadequacy of ICT policies) is crucial as the first knowledge base towards improving implementation of any evidence-based intervention (EBI)^18–21,25^, understanding its ICD is equally important to effectively guide implementation efforts^25,26^. Specifically, implementers use the knowledge gained from ICD of EBI to inform the selection of appropriate strategies needed for its effective implementation and public health outcomes^27^.

In the few studies where implementation of e-learning has been deemed successful in the public health sector in Africa, findings suggest that its implementation contexts are mostly linked to constraints or enabling factors around inner setting (public health institutions), outer setting (governance systems), and process (administrative procedures)^10,22–24^. Evidence from the same studies also indicates implementation determinants related to training resource availability, training contents adaptability, training design, access to ICT knowledge and information, and supportive policies and laws^10,22–24^. However, the relationships between e-learning ICD suggested in these studies remain unclear, in part, due to a lack of systematic analysis of identified constraints or success factors through the implementation science lens. Elucidating the connections between these contexts and determinants using sound theoretical principles could help to better understand the underlying processes associated with implementation success or failure of e-learning. This understanding could guide its implementation for global health security competency-based trainings (e.g., EPR) among the public health workforce in a more systematic, practical, and effective manner^28,29^.

This study aims to (1) identify MOT implementation contexts, (2) assess MOT implementation determinants, and (3) characterize the relationships between MOT implementation contexts and determinants among the public health workforce in West Africa using the updated Consolidated Framework for Implementation Research (CFIR).

## Results

### Implementation contexts of MOT

Intervention (26.7%) was the most frequently identified implementation contexts of MOT, followed by inner setting (23.3%), outer setting (23.3%), process (20.0%), and individuals (6.7%), as indicated in Table 1.

**Table 1:** Implementation contexts, determinants, and relationships of multi-communication online training (MOT) among public health workers in West Africa based on CFIR, from August 2023 to October 2023^30^.

Characteristic	MOT Constraint *N* = 15	MOT Enabler *N* = 15	Total *N* = 30
	*n*	*n*	*n* (%)
Context			
Intervention	4	4	8 (26.67)
Individuals	1	1	2 (6.67)
Inner setting	4	3	7 (23.33)
Outer setting	3	4	7 (23.33)
Process	3	3	6 (20.00)
Determinant			
Design	2	3	5 (16.67)
Policies & laws	2	2	4 (13.33)
Available resources	2	1	3 (10.00)
Local conditions	1	2	3 (10.00)
Capability	1	1	2 (6.67)
Assessing needs	1	1	2 (6.67)
Engaging	1	1	2 (6.67)
Cost	1	0	1 (3.33)
Complexity	1	0	1 (3.33)
Structural characteristics	1	0	1 (3.33)
Communications	1	0	1 (3.33)
Planning	1	0	1 (3.33)
Adaptability	0	1	1 (3.33)
Access to knowledge & information	0	1	1 (3.33)
Relational connections	0	1	1 (3.33)
Adapting	0	1	1 (3.33)
Relationship			
*Intervention*	*N* = 4	*N* = 4	*N* = 8
Design	2	3	5 (62.5)
Adaptability	0	1	1 (12.5)
Complexity	1	0	1 (12.5)
Cost	1	0	1 (12.5)
*Individuals*	*N* = 1	*N* = 1	*N* = 2
Capability	1	1	2 (100)
*Inner setting*	*N* = 4	*N* = 3	*N* = 7
Available resources	2	1	3 (42.9)
Structural characteristics	1	0	1 (14.3)
Communications	1	0	1 (14.3)
Access to knowledge & information	0	1	1 (14.3)
Relational connections	0	1	1 (14.3)
*Outer setting*	*N* = 3	*N* = 4	*N* = 7
Policies & laws	2	2	4 (57.1)
Local conditions	1	2	3 (42.9)
*Process*	*N* = 3	*N* = 3	*N* = 6
Assessing needs	1	1	2 (33.3)
Engaging	1	1	2 (33.3)
Planning	1	0	1 (16.7)
Adapting	0	1	1 (16.7)

Percentage distribution of implementation determinants sums to 99.9% due to approximation.

### Implementation determinants of MOT

A total of 16 implementation determinants of MOT were identified. The most common implementation determinants of MOT identified were design (16.7%), policies and laws (13.3%), available resources (10.0%), local conditions (10.0%), capability (6.67%), assessing needs (6.67%), and engaging (6.67%) while cost, complexity, structural characteristics, communications, planning, adaptability, access to knowledge and information, relational connections, and adapting (3.3% each) were the least common determinants, as shown in Table 1.

### Relationships between implementation contexts and determinants of MOT

**Fig. 1:**
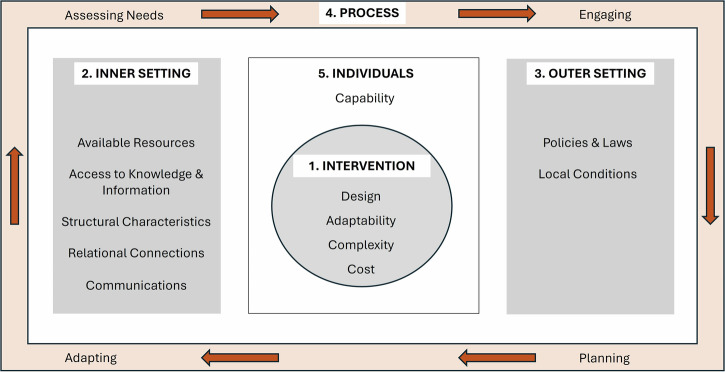
A ranked relational framework showing empirical relationships between implementation contexts and determinants of multi-communication online training (MOT) among public health workers in West Africa based on CFIR, from August 2023 to October 2023^30^. The *numbered* ranking of the intervention, individuals, inner setting, outer setting, and process contexts denotes the “priority level of implementation efforts and resources for contexts” and not the “order of implementation actions for contexts” based on their proportions in Table 1 (context), given that all contexts are expected to have their implementation actions carried out simultaneously. Implementation determinants in the intervention, individuals, inner setting, and outer setting contexts are ranked in a *top-down descending* priority level of implementation efforts and resources, while determinants in the process context are ranked in a *sequential* priority level of implementation efforts and resources and order of implementation actions based on their proportions in Table 1 (relationship). The unidirectional arrows in the process context denotes an iterative non-causal relationship, showing a step-by-step guidance for which determinant is to be prioritized during implementation before the other. For example, for MOT implementation context, more implementation efforts and resources (e.g., time, funds) should be devoted to individuals, followed by process, outer setting, inner setting, and intervention. For its process context, assessing needs determinant (step 1), is followed by engaging (step 2), planning (step 3), and adapting (step 4), with the cycle repeated, as needed. For its intervention, individuals, inner setting, and outer setting contexts, more implementation efforts and resources should be devoted to design, capability, available resources, and policies and laws determinants, respectively, and followed by their next determinants.

Based on CFIR^30^, Fig. 1 shows the empirical relationships of the MOT implementation determinants in their varying contexts and how they may be targeted with appropriate strategies and level of effort and resources to influence successful MOT implementation outcomes. In line with CFIR and the study team’s field experience, the main assumption underlying the relationships of MOT ICD described in this study is that concurrent interactions among all implementation contexts and their determinants are often necessary to influence an implementation outcome. As encouraged by The Lancet Global Health^31^, we extended the application of CFIR in this study for more practical utility based on real-world evidence. Given the preponderance of MOT implementation challenges, including time constraints and budget limitations similar to other EBI in a real-world setting, the relationships of MOT ICD are ranked in a *numbered* or *top-down descending*, or *sequential* priority level of implementation effort and resources, as well as order of implementation actions, as appropriate based on their proportions in Table 1 (context & relationship).

## Discussion

Leveraging data from a multi-country mixed-methods study on MOT constraints and enablers and the CFIR theoretical framework^30,32^, for the first time to our knowledge, this study identified key MOT ICD and characterized their relationships to inform successful implementation among the public health workforce for global health security competency-based trainings in a LMICs setting. MOT ICD found in this study were representative across all the five CFIR domains for EBI implementation, including intervention, individuals, inner setting, outer setting, and process though with variable relative importance (i.e., proportions)^30^. Findings from this study demonstrate that it is crucial to understand the ICD of e-learning in its entirety using robust evidence and relevant theories or frameworks (e.g., CFIR) to systematically identify critical aspects of an e-learning implementation ecosystem for scalable changes towards improved implementation and public health outcomes^17,30,31^.

This study findings are consistent with other studies conducted among public health workers in Liberia, Kenya, South Africa, and across the African continent^10,23,24,33^. Interestingly, our study found intervention as the most important element of e-learning implementation contexts within the public health workforce other than inner setting as previously suggested in the literature^10,22–24^. This new insight might explain the evidence from a recent study among public health workers in West Africa, which showed that the contextual fit of e-learning is suboptimal^32^. This finding is not surprising because multiple studies have shown that the implementation of EBIs (e.g., MOT) in a majority of under-resourced settings like Africa is usually unsuccessful, in part, due to the failure of implementers in adequately adapting EBIs to their local contexts before their implementation and scale-up^15,17,22,34,35^. Thus, this study’s findings highlight the need for implementers to focus first on the e-learning intervention by assessing its “core functions” (i.e., key components of an EBI that are central to the achievement of its intended goals) and “forms” (i.e., activities required to perform each component of an EBI) to explore potential areas for its adaptation across design and delivery based on contextual realities and needs^36,37^.

Unlike other studies^10,23,24,33^, this study further showed the relative importance of other implementation contexts of MOT with inner and outer settings closely following intervention, then process, and lastly individuals. Notably, the equal proportions of inner setting and outer setting as revealed in this study suggest that similar implementation efforts and resources may need to be given to both settings to successfully implement e-learning interventions for global health security competency-based trainings (e.g., EPR) among the public health workforce in West Africa^28,29^. This additional insight from this study is contrary to what evidence from the field suggests. Traditionally, implementation efforts and resources on EBIs, including e-learning are largely directed at the inner setting with minimal or ad hoc efforts and resources expensed on the EBI implementation issues at the outer setting^10,23,33,38^. For instance, at the national level of the inner setting, Walsh and colleagues from Liberia reported on how the implementation of their e-learning intervention for medical training was challenging due to unregulated internet costs and the lack of existing platforms in the government or civil society to lead necessary policy change efforts^33^.

Similarly, while there are increasing technical and financial investments for improved implementation of e-learning for global health security competency-based trainings at the regional level of inner settings such as the Africa Centers for Disease Control and Prevention (CDC) Institute for Workforce Development^39^, disparities in ICT investments by African governments in ensuring availability of local resources (e.g., stable electricity, quality and affordable internet) continue to reduce e-learning’s reach, effectiveness, and associated public health impact^5,18,40^. Therefore, to address these challenges, public health practitioners must pursue more authentic partnerships with key government sectors (e.g., economic, ICT) at the national, regional, and global levels from exploration and planning through the execution phases of e-learning implementation. Further, this study demonstrated the pertinent role that process and individuals play in e-learning implementation. To maximize the impact of e-learning implementation and associated public health outcomes, we argue that implementation activities targeted at the process and individual levels need to be evidence-informed, culturally responsive, systematic, and continuous across all implementation stages. Overall, understanding the key implementation contexts of e-learning and their level of importance could help implementers to prioritize implementation efforts and resources. This is particularly relevant in settings like Africa, where constraints to e-learning implementation are enormous and resources are not only limited but also have a higher likelihood of being poorly managed^18,32,41^.

This study found design as the leading determinant of MOT implementation overall and at the intervention level. This study finding agrees with other studies conducted in Africa and even globally^10,15,22–24,42^. For example, a study by Thomas and colleagues from Nigeria suggested that the implementation of their e-learning intervention for health professionals using a learner-centered design that involved its packaging and delivery as a “modular, self-paced, mobile-ready, and work-relevant” intervention was a key factor for its success^42^. This study finding is also similar to another study by Kessy and colleagues that evaluated the implementation of an infection prevention and control e-learning intervention for health workers across the African continent during the COVID-19 pandemic, which found that the use of multiple ICT-enabled approaches, including social media learning, multi-language translations, webinar series, and online community of practice contributed immensely to its successful implementation with high reach and satisfactory learning outcomes^10^. In fact, two different global studies conducted in 2013 and 2023 consistently found design as a major success factor of e-learning intervention in health-related fields^15,22^. Findings from these studies also align with other MOT intervention-level implementation determinants, including adaptability, complexity, and cost identified in this study. These findings indicate that implementers must engage with public health workers to iteratively assess their preferences, needs, and priorities to ensure the delivery of a contextually fit e-learning intervention on global health security competencies for better implementation and public health outcomes.

Surprisingly, we found that policy and laws accounted for the second top implementation determinant of MOT, which is closely followed by local conditions at the outer setting level. These findings show that directing appropriate actions with proportionate implementation efforts and resources towards implementation determinants at the outer setting in tandem with other contexts’ determinants may be necessary to successfully implement e-learning interventions on global health security in under-resourced settings. Further, it can be plausibly assumed that favorable policies and laws and investments within the power, ICT, and health sectors would result in significant improvement of local conditions such as reliable electricity, high internet bandwidth, and better working conditions (e.g., fair remuneration) that are needed to ensure the accessibility and sustained motivation of public health workers for e-learning interventions on global health security competencies^18–20^. In addition to the many ongoing government initiatives (e.g., rural digital technology expansion in Benin, 5 G internet deployment in Mali) that directly or indirectly address some of these identified implementation issues^43,44^, the establishment of civil society-led advocacy groups such as the National Research and Education Network (NREN) in Sierra Leone^45^, which engages with internet network providers in collaboration with the government and relevant stakeholders for reduced internet costs, could contribute to the accelerated progress of e-learning implementation for global health security competency-based trainings in the region, if replicated by other African countries.

More so, this study found available resource as the third overall leading implementation determinant of MOT and the top determinant at the inner setting level in addition to access to knowledge and information, structural characteristics, relational connections, and communications. These findings shed more light on some of the documented practical measures, including but not limited to internet data incentivization, massive open online courses delivery, multi-communication feedback system, and online trainer-trainee mentorship that are used for addressing issues around these identified determinants during implementation of e-learning interventions in the public health workforce in Africa and other similar under-resourced settings. Though while these efforts are laudable, their quality and sustainability are unequal within and between settings due to existing digital and economic disparities^40,46^. Hence, this highlights the need for “whole-of-society” approach across research, advocacy, and policymaking to effectively and efficiently address these e-learning implementation issues.

In this study, capability, assessing needs, and engaging tied as the fourth top implementation determinants of MOT. These findings are consistent with the existing body of knowledge^10,15,22–24,42^. Concerning capability, it is recommended that for e-learning interventions to be successful, they should be delivered with the provision of a basic digital literacy training for both participants and trainers to address general and specific needs that may hinder effective participation and retention in the training^7,47^. Also, this study findings on assessing needs and engaging are supported by practices in the field such as pre and post training surveys and stakeholder analysis and mapping to ensure that resources are for e-learning implementation are not only mobilized but are also directed towards essential needs of the trainees. These determinants and other findings on cost, complexity, structural characteristics, adaptability, access to knowledge and information, and relational connections identified in this study are in agreement with previously studies, further highlighting the complexity of e-learning implementation in an LMIC setting like Africa.^18–20^

Overall, findings from this study underscore the importance of understanding the ICD of e-learning in the public health workforce in a theoretically informed and practical manner given the complexity of its implementation issues. This information is fundamental to any e-learning implementation efforts by public health manager, trainers, and policymakers in collaboration with academics. This study’s findings provide baseline evidence and approach for informing the selection and prioritization of implementation strategies using well-established implementation research methods such as the CFIR-Expert Recommendation for Implementation Change (ERIC) matching system to guide the development of an e-learning implementation protocol for implementers in West Africa and similar settings^27^. More importantly, our findings suggest that proportionate priority level of implementation efforts and resources may be needed to systematically carry out e-learning implementation strategies and actions for its successful implementation in global health security competency-based trainings among the public health workforce in under-resourced settings. These insights align with ongoing digital transformation efforts in LMICs such as the African Union’s Digital Transformation Strategy for Africa 2020–2030. This strategy is reassuring, as it prioritizes investments for some of the identified MOT constraints and enablers, particularly in the areas of providing affordable and fast internet connectivity, increased basic digital literacy training across sectors including health, and implementation of inclusive digital policies and laws at the institutional, national, and regional levels^48^.

This study has some strengths and limitations. One of the strengths of this study is the use of a mixed-methods design and subregional data to enhance the robustness and representativeness of findings. Additionally, the analysis of this study data with a team-based coding approach as the gold standard for this study rather than individual coding likely improved the reliability of data coding for this study findings. Lastly, unlike most studies, this study extended the use of CFIR to demonstrate the empirical relationships of implementation contexts and determinants, which could further support its real-world application for implementation of e-learning interventions in under-resourced settings.

Despite these strengths, the findings in this study should be interpreted with the consideration of its limitations. First, this study findings are subject to the inherent limitations of its underlying data, including selection bias posed by its non-random sampling technique, variability of participating countries’ sample size, unbalanced perspectives of interviewees, and social desirability bias, which may all contribute to reduced representativeness to West Africa and other LMICs. However, efforts were made to promote widespread and intensive dissemination of the study through multiple communication channels, including One Health forum, emails, institutional newsletters, and social media to ensure the selection of a diverse group of participants, in improving the study accuracy and representativeness. Second, we did not perform any subgroup analysis due to a small sample size. This would have provided a nuanced understanding of the distribution and relationships of MOT constraints and enablers across key subgroups (e.g., discipline category, health sectors, work area, country income group) with potentially different social, economic, and political conditions. Third, we did not report the specific characteristics (i.e., subconstructs) of some implementation determinants, including available resources, assessing needs, and engaging implementation determinants, thus resulting in an incomplete understanding of these determinants. Fourth, this study findings have a potential for information bias from non-differential misclassification of the MOT constraints and enablers due to potential differences in cognitive judgments of the study team because of unequal implementation science competencies. Fifth, the distribution and relationships of MOT ICD described in this study based on CFIR, may not provide a complete picture of the contexts and determinants associated with MOT implementation. This is because other important constructs (e.g., behavioral dynamics, scale-up processes) theorized in other implementation science frameworks or those completely missed in CFIR and other frameworks (e.g., power dynamics, competing interests) were excluded. Sixth, the generalizability of this study findings to West Africa is also limited due to secular changes in the MOT constraints and enablers from the time the data underlying this study were collected. Seventh, the distribution and relationships of MOT ICD characterized in this study are only descriptive and do not demonstrate any confirmed associations or relationships and causal effects. Eight, this data-driven ranked relational framework is sensitive to changes in the set of constraints and enablers identified for coding and their proportional distribution based on the study context by geographic setting, study population, and time period (i.e., a different set of constraints and enablers from another study context would result in different proportions). Nineth, the IDI guide in the initial study from which MOT constraints and enablers were enumerated was not piloted, which may have resulted in inconsistent interview practice and limited depth of the data collected. While this was the case, measures including expert review, the use of experienced interviewers, and thick description of transcripts were taken to mitigate these potential weaknesses, suggesting that the final data still provided rich insights. Tenth, while the team-based coding employed in this study may have likely improved the reliability of findings, it may have also introduced conformity bias, where opposing coders follow the majority view during consensus discussions. Further research with inferential analysis of data from observational (e.g., cross-sectional) or experimental (e.g., randomized controlled trial) study is needed to establish such interpretations and conclusions. Nevertheless, this study adds to the relatively small proportion of studies that have used CFIR to comprehensively and systematically describe the constraints and enablers to e-learning among the public health workforce. This study findings could help to deepen the understanding of the contexts and determinants associated with the implementation of e-learning for global health security competency-based trainings in an African setting.

To advance our current understanding of e-learning implementation, future quantitative research should consider establishing confirmed associations or relationships between implementation contexts and determinants in addition to their causal effects on implementation success. Implementation success should be defined and measured alongside implementation context and determinants as categorical variables. For example, study participants can be asked questions with a “Yes” or “No” response on their perceived relevance of CFIR domains (i.e., implementation contexts), EBI constraints and enablers (i.e., implementation determinants), and EBI predefined constructs (e.g., effectiveness, contextual fit, and feasibility) of implementation success based on their individual or institutional experiences. This is crucial to make findings from this study as *a priori framework* or those from CFIR falsifiable. This can be achieved irrespective of the planned utility of these frameworks, either as a guide or as a tool development and validation for the assessment of these implementation outcomes. More details can be found in the future directions of the Supplementary Material.

In conclusion, we systematically identified the ICD of e-learning in the limited background of this study. We also demonstrated their empirical relationships through a data-driven relational framework based on CFIR to better understand the complexity of e-learning implementation challenges, thereby suggesting practical ways for change actions in a resource-efficient manner. Thus, this study’s findings could be used by implementers, including researchers, practitioners, managers, trainers, and policymakers to inform the design of evidence-based strategies and prioritization of efforts and resources for building the capacity of the public health workforce in under-resourced settings on core competencies such as EPR. Given the differences in the depth and pace of technological and socioeconomic development within and between countries, this study provides a novel approach that can be replicated and validated for consistent assessment of e-learning ICD for scalable implementation change efforts. Finally, this study contributes to the advancement of implementation science in e-learning research and practice for real-world health impact.

## Methods

### Study design

This was a multi-country, cross-sectional study. We performed a secondary analysis of triangulated quantitative and qualitative data from a previous larger mixed-methods study that assessed the contextual fit and feasibility of MOT as well as its constraints and enablers to inform the implementation of global health security competency-based trainings^32^. The purpose of this present study is to identify MOT ICD that might influence the outcomes (success or failure) of its implementation.

### Study settings

This study was conducted in 16 West African countries, representing English, French, and Portuguese speaking low-and middle-income countries (LMICs) settings. These included Gambia, Ghana, Guinea, Liberia, Nigeria, and Sierra Leone for *English* setting; Benin, Burkina Faso, Cote d’Ivoire, Mali, Mauritania, Niger, Senegal, and Togo for the *French* setting; and Cape Verde and Guinea-Bissau for the *Portuguese* setting.

### Study population and sampling

This study was approved by the Emory University Institutional Review Board (NHSR Determination Form, 3/22/23), and the West African Health Organization (WAHO) provided institutional authorization for its conduct. The study population that contributed data to this present study included a non-random sample of public health workers across the human, animal, and environmental health sectors in the 16 West African countries, who participated in the above stated larger study between Aug 10, 2023 and Oct 10, 2023^32^. The quantitative component of the larger study included 237 participants of an estimated sample size of 222 public health workers given an expected proportion of 90.5% of public health workers that considered e-learning as an acceptable modality of training in an African setting at 95% confidence level, and after accounting for a non-response rate of 40% for most online surveys specific to e-learning^32^. Participants were representative of three broad categories: core public health workers (CPHW), healthcare workers with one or more public health functions (HW-PH), and allied workers (AW). CPHW were those who identify their work discipline as public health specialists. HW-PH included physicians, nurses, laboratory scientists, and laboratory technicians. AW comprised of environmental health scientists, environmental health technicians, veterinarians, and assistant veterinarians^32^. The qualitative component included seven interviewees, representing public health managers, trainers, and policymakers. Quantitative and qualitative components had female-male ratios of approximately 1:2 and 2:1, respectively. A rural-urban ratio of 1:2 was reported for the quantitative component, but this was 1:1 for the qualitative component with a mixed picture, where each interviewee indicated having work experience in both rural and urban areas prior to the study. The study participants were selected via a virtual snowball technique and provided quantitative data on MOT constraints and enablers through an online survey, while interviewees were selected via a voluntary response technique and provided qualitative data through an in-depth interview (IDI)^32^.

### Data collection and tools

In the larger study, a self-administered semi-structured online questionnaire was employed for quantitative data collection, while a semi-structured IDI guide was used for qualitative data collection. The development of the questionnaire and IDI guide were informed by literature^32^. Specific to this present study outcome of interest, the questionnaire contained a closed-ended, multiple-response question on 10 MOT constraints and 5 MOT enablers, plus an open-ended question on “Others” considered by the participants. The IDI guide had main and probing open-ended questions. The questionnaire was pilot tested, but the IDI guide was not due to sample size and time constraints. Both questionnaire and IDI guide were available in multiple languages, with their development involving two face validity improvement processes. These included peer review and feedback incorporation from: 1) the study team, and 2) 43 public health experts from a subregionally coordinated One Health forum. These were achieved through multiple iterations of physical and online discussions leveraging one-on-one, email, and Zoom communication platforms. Specifically, quantitative data on MOT constraints and enablers were collected from the study participants via Google Form weblink, while qualitative data were collected via Zoom. Quantitative and qualitative data were then descriptively analyzed and thematically coded, respectively^32^. Informed consents were collected for both survey and IDI, with data confidentiality ensured throughout the study.

### Theoretical framework

In this present study, we used the updated CFIR as the overarching framework to demonstrate the theoretical underpinning of this study given its extensive utility and comprehensiveness^30,49^. Each identified constraint and enabler to MOT was coded onto the most appropriate CFIR domains and constructs for the identification of MOT ICD, respectively, and ultimately to characterize their relationships.

As presented in Table 2, CFIR is a comprehensive meta-framework with synthesized factors that may hinder or contribute to the implementation success of an EBI from a wide range of implementation theories, models, and frameworks into a single structure^30,49^. It is used for the systematic assessment of potential constraints and/or enablers of an EBI. It provides a structured approach for assessing the constraints and enablers of an EBI prospectively or retrospectively. It is used as a guide or as a basis for tool development and validation to systematically assess EBI constraints and enablers across five domains (intervention, individuals, inner setting, outer setting, and process) and 39 constructs to identify determinants that might influence their implementation outcomes^30^. Findings from this exercise can then be used to inform the selection of implementation strategies to mitigate the constraints and leverage the enablers. While the original CFIR is a widely used determinant framework for implementation research^49^, the updated CFIR was chosen for this analysis because it provided a clearer and consistent description of its constructs for improved applicability^30^.

**Table 2:** CFIR domains, constructs, and definitions^30^.

CFIR Domain	Construct	Definition
		*The degree to which:*
Intervention (The “thing” being implemented, e.g., a new clinical treatment, educational program, or city service)	Source	The group that developed and/or visibly sponsored use of the intervention is reputable, credible, and/or trustable.
	Evidence-Base	The intervention has robust evidence supporting its effectiveness.
	Relative Advantage	The intervention is better than other available interventions or current practice.
	Adaptability	The intervention can be modified, tailored, or refined to fit local context or needs.
	Trialability	The intervention can be tested or piloted on small scale and undone.
	Complexity	The intervention is complicated, which can be reflected by its scope and/or the nature and number of connections and steps.
	Design	The intervention is well designed and packaged, including how it is assembled, bundled, and presented.
	Cost	The intervention purchase and operating costs are affordable.
Individuals *Characteristics* (The importance of the characteristics of individuals)	Need	The individual(s) has deficits related to survival, well-being, or personal fulfillment, which may be addressed by implementation and/or delivery of the intervention.
	Capability	The individual(s) has interpersonal competence, knowledge, and skills to fulfill role.
	Opportunity	The individual(s) has availability, scope, and power to fulfil role.
	Motivation	The individual(s) is committed to fulfilling role.
Inner setting (The setting in which the intervention is implemented, e.g., hospital, school, city)	Structural Characteristics	Infrastructure components support functional performance of the inner setting.
	Relational Connections	There are high quality formal and informal relationships, networks, and teams within and across Inner Setting boundaries (e.g., structural, professional).
	Communications	There are high quality formal and informal information sharing practices within and across Inner Setting boundaries (e.g., structural, professional).
	Culture	There are shared values, beliefs, and norms across the Inner Setting.
	Tension for Change	The current situation is intolerable and needs to change.
	Compatibility	The intervention fits with workflows, systems, and processes.
	Relative Priority	Implementing and delivering the intervention is important compared to other initiatives.
	Incentive Systems	Tangible and/or intangible incentives and rewards and/or disincentives or punishments support implementation and delivery of the intervention.
	Mission Alignment	Implementing and delivering the intervention is in line with the overarching commitment, purpose, and goals in the Inner Setting.
	Available Resources	Resources are available to implement and deliver the intervention.
	Access to Knowledge & Information	Guidance and/or training is accessible to implement and deliver the intervention.
Outer setting		
(The setting in which the Inner Setting exists, e.g., hospital system, school district, state)	Critical Incidents	Large-scale and/or unanticipated events disrupt implementation and/or delivery of the intervention.
	Local Attitudes	Sociocultural values (e.g., shared responsibility in helping recipients) and beliefs (e.g., convictions about the worthiness of recipients) encourage the Outer Setting to support implementation and/or delivery of the intervention.
	Local Conditions	Economic, environmental, political, and/or technological conditions enable the Outer Setting to support the implementation and/or delivery of the intervention.
	Partnerships & Connections	The Inner Setting is networked with external entities, including referral networks, academic affiliations, and professional organization networks.
	Policies & Laws	Legislation, regulations, professional group guidelines and recommendations, or accreditation standards support implementation and/or delivery of the intervention.
	Financing	Funding from external entities (e.g., grants, reimbursement) is available to implement and/or deliver the intervention.
	External Pressure	External pressures drive implementation and/or delivery of the intervention.
Process (The activities and strategies used to implement the intervention)	Teaming	Join together, intentionally coordinating and collaborating on interdependent tasks, to implement the intervention.
	Assessing Needs	Collect information about priorities, preferences, and needs of people.
	Assessing Context	Collect information to identify and appraise barriers and facilitators to implementation and delivery of the intervention.
	Planning	Identify roles and responsibilities, outline specific steps and milestones, and define goals and measures for implementation success in advance.
	Tailoring Strategies	Choose and operationalize implementation strategies to address barriers, leverage facilitators, and fit context.
	Engaging	Attract and encourage participation in implementation and/or the intervention.
	Doing	Implement in small steps, tests, cycles of change to trial and cumulatively optimize delivery of the intervention.
	Reflecting & Evaluating	Collect and discuss quantitative and qualitative information about the success of implementation.
	Adapting	Modify the innovation and/or the Inner Setting for optimal fit and integration into work processes.

Table 2 (cont’d). *CFIR* Consolidated framework for implementation research. Note: “intervention” is also known as “innovation”.

In this study, we operationalized CFIR retrospectively and as a guide. We considered this appropriate to avoid any potential selection bias that may occur from the study team’s prioritization of CFIR constructs when used prospectively and as a tool development and validation, to improve its real-world utility. We followed two steps to identify MOT ICD. These included collation of secondary data on MOT constraints and enablers from the larger study and deductive coding of each MOT constraint and enabler onto the most appropriate CFIR domain and construct, as described below.

### Data preprocessing

**Fig. 2:**
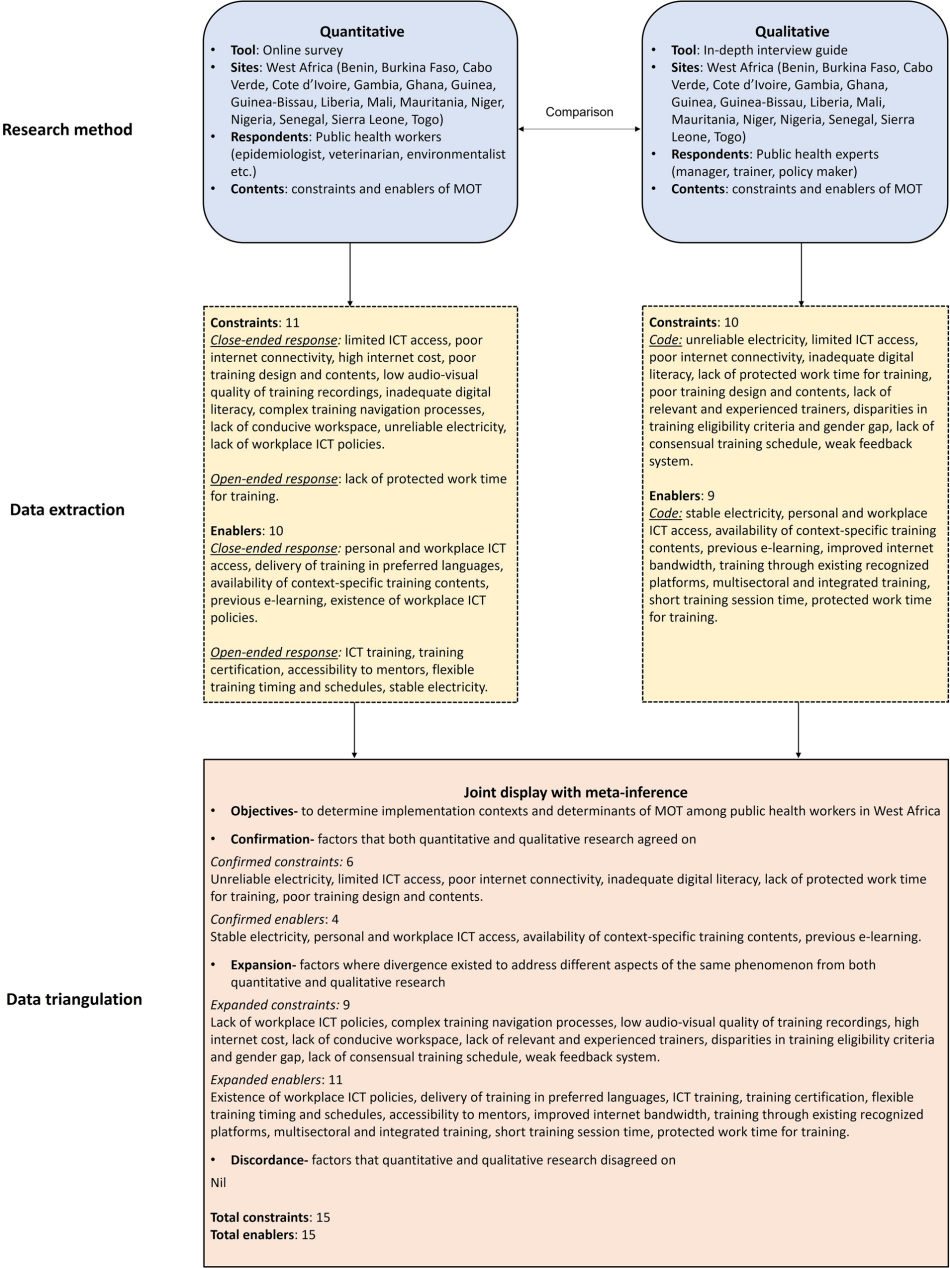
Summary of the research method, data extraction, and data triangulation. *MOT* Multi-communication online training, *ICT* Information and communication technology^32^.

In step 1, we collated secondary data on MOT constraints and enablers. Quantitative and qualitative data on MOT constraints and enablers from the previously conducted larger study were extracted. Quantitative data extracted had 11 MOT constraints (*closed-ended response*: 10, *open-ended response*: 1) and 10 enablers (*closed-ended response*: 5, *open-ended response*: 5), while qualitative data provided 10 MOT constraints and 9 enablers. Thereafter, data were triangulated using a joint display approach^32^, and then entered into Microsoft Excel. The joint display approach allows the identification of themes or variables across three meta-inferences, including: confirmation (i.e., agreement between quantitative and qualitative data), expansion (i.e., identification of different aspects of the same phenomenon observed in quantitative and qualitative data, and discordance (i.e., disagreement between quantitative and qualitative data). A total of 30 observations (15 each for constraints and enablers) were entered, and additional data fields were created for ICD based on CFIR^30^. More details on the research method, data extraction, and triangulation can be found in Fig. 2.

### Data analysis

In step 2, we performed deductive coding of MOT constraints and enablers onto CFIR domains and constructs. To identify MOT ICD, its constraints and enablers were deductively coded using a codebook developed based on CFIR domains and constructs, as shown in Table 2^30^. We first performed an individual coding and then a team-based coding to reduce information bias from potential cognitive misjudgements from the initial coders for improved accuracy of the final codes. For the individual coding, we purposively selected two members of the study team, who had recent experiences with the application of CFIR in their respective practices. Both individuals refamiliarized themselves with the CFIR domains and constructs using the study codebook and sought clarifications on the meanings of terminologies among their peers where necessary. The two coders were individually provided with a checklist (Supplementary Table 1) containing the information on the MOT constraints and enablers and were required to independently code each MOT constraint and enabler onto the most appropriate CFIR construct and domain. Identified codes were then shared amongst them. Disagreements were easily resolved through one-on-one discussion to produce the initial codes for MOT ICD, which were then finalized through team-based coding. In the team-based coding, all team members jointly coded the MOT constraints and enablers using the study codebook and the CFIR framework guide until consensus was reached by the endorsement of codes with the highest proportion for each observation. All team members validated the final codes for analysis, as mapped in Tables 3 and 4. The final codes were entered into the Microsoft Excel dataset and then imported into SPSS version 29 for descriptive analysis.

**Table 3:**
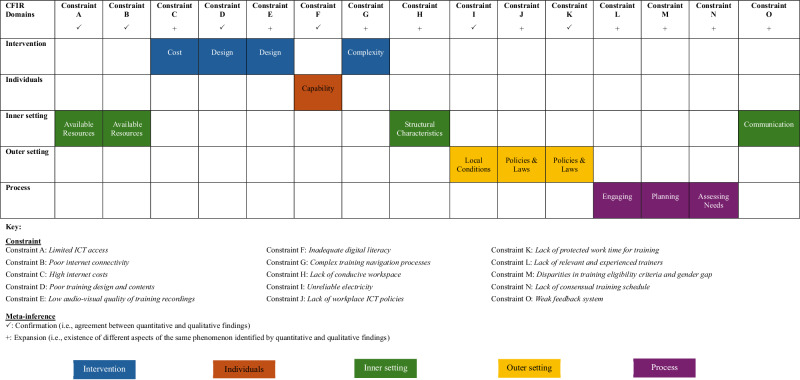
Mapping of multi-communication online training (MOT) constraints among public health workers in West Africa reported from August 2023 to October 2023 to identify its implementation contexts and determinants using CFIR^30^.

*MOT* Multi-communication online training, *CFIR* Consolidated framework for implementation research. Note**:** constraints are unranked.

**Table 4:**
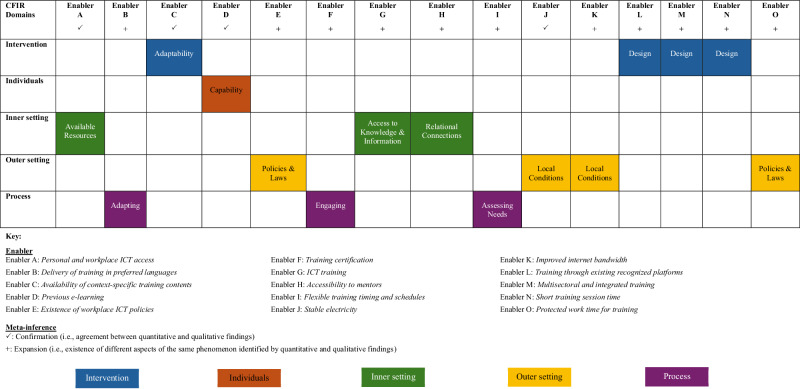
Mapping of multi-communication online training (MOT) enablers among public health workers in West Africa reported from August 2023 to October 2023 to identify its implementation contexts and determinants using CFIR^30^.

*MOT* Multi-communication online training, *CFIR* Consolidated framework for implementation research. Note**:** enablers are unranked.

As a decision rule on how ambiguous cases could be resolved should they be encountered during individual and team coding, authors were required to engage in (1) individual or group literary analysis of implementation constraints and enablers to assess the relationships between the syntactics (word structure) and semantics (word meaning) of their phrases, and (2) combine this literary analysis insights with the contextual knowledge of the implementation ecosystem of the evidence-based intervention and geographic setting under study, to ensure that the coding reflects a real-world implementation as much as possible. For instance, in the case of constraint I: “high internet costs” in Table 3, the word “internet” acts as a noun adjunct or adjective-noun, qualifying “costs”. More so, regarding any e-learning interventions like MOT, the authors have the experiential understanding that internet is the most specific resource that this type of intervention would directly depend upon to be implemented. Hence, making its coding under intervention’s cost construct the most appropriate and uncontested between the individual coders and the team-based codings. On the other hand, if this was to be other equally important resources such as “technology personnel costs” or “electricity costs”, the former would have been coded under inner setting’s available resources and the latter under outer setting’s local conditions.

To assess the reliability of data coding, we performed two intercoder reliability tests with percent agreement and Cohen’s kappa statistics on R version 4.3.0. For both statistics, the first reliability test was conducted between the first coder and second coder (Supplementary Table 2, Supplementary Figs. 1–3), while the second reliability test was between the initial coding and final coding (Supplementary Table 3, Supplementary Figs. 4–6). Between the first coder and the second coder, we found a percent agreement of 83.3% (constraints: 80.0%, enablers: 86.7%) and a Cohen’s kappa of 0.82 (constraints: 0.78, enablers: 0.85). Additionally, between the initial coding and the final coding, we found a percent agreement of 86.7% (constraints: 86.7%, enablers: 86.7%) and a Cohen’s kappa of 0.85 (constraints: 0.85, enablers: 0.85).

## Supplementary information

Supplementary information

## Data Availability

The data underlying this article are available at 10.6084/m9.figshare.24654486.v8.

## Code availability

The relevant codes for this study are available at https://github.com/Kogunye/MOT-Contexts-and-Determinants.git.
