# Supplementary material for: Understanding implementation contexts and determinants of e-learning for global health security competency-based training in LMICs
Source: Npj Health Syst. 2026 Jul 1;3:52. doi: 10.1038/s44401-026-00105-z (PMC13354242; doi:10.1038/s44401-026-00105-z)
Supplement: Supplementary file 1 — Supplementary information [file 44401_2026_105_MOESM1_ESM.pdf]

Understanding implementation contexts and determinants of e-learning for global health security competency-based training in LMICs

Supplementary Information

Table of contents

Supplementary Table 1: Data coding checklist…………………………………………………………...1

Supplementary Table 2: First intercoder reliability (percent agreement statistics)………………………2

Supplementary Table 3: Second intercoder reliability (percent agreement statistics)……………………3

Supplementary Figures 1-3: First intercoder reliability (Cohen's kappa statistics)...........………………4-7

Supplementary Figures 4-6: Second intercoder reliability (Cohen's kappa statistics)…….……………..8-11

Supplementary Discussion: Future directions………………………………………………………………12

References….……………………………………………………………………………………………13

Supplementary Table 1: Data coding checklist

Instruction

- Choose and indicate the most appropriate CFIR construct with its domain for each multi-communication online training (MOT) constraint provided below based on the study codebook

Coding key:

CFIR construct and domain[1]

Intervention: Source= SO, Evidence-base= EV, Relative advantage= RA, Adaptability= AD, Trialability= TR, Complexity= CO, Design= DE, Cost= CS

Individuals: Need= NE, Capability= CA, Opportunity= OP, Motivation= MO

Inner setting: Structural characteristics= SC, Relational connections= RC, Communications= COM, Culture= CU, Tension for change= TC, Compatibility= CP, Relative priority= RP, Incentive systems= IS, Mission alignment= MA, Available resources= AR, Access to knowledge & information= AKI

Outer setting: Critical incidents= CI, Local attitudes= LA, Local conditions= LC, Partnerships & connections= PC, Policies & laws= PL, Financing= FI, External pressure= EP

Process: Teaming= TE, Assessing needs= AN, Assessing context= AC, Planning= PA, Tailoring strategies= TS, Engaging= EN, Doing= DO, Reflecting & evaluating= RE, Adapting= AA

S/N	MOT Constraint	CFIR Construct	CFIR Domain
1	Limited ICT access		
2	Poor internet connectivity		
3	High internet costs		
4	Poor training design and contents		
5	Low audio-visual quality of training recordings		
6	Inadequate digital literacy		
7	Complex training navigation processes		
8	Lack of conducive workspace		
9	Unreliable electricity		
10	Lack of workplace ICT policies		
11	Lack of protected work time for training		
12	Lack of relevant and experienced trainers		
13	Disparities in training eligibility criteria and gender gap		
14	Lack of consensual training schedule		
15	Weak feedback system		
	**MOT Enabler**		
1	Personal and workplace ICT access		
2	Delivery of training in preferred language		
3	Availability of context-specific training contents		
4	Previous e-learning		
5	Existence of workplace ICT policies		
6	Training certification		
7	ICT training		
8	Accessibility to mentors		
9	Flexible training timing and schedules		
10	Stable electricity		
11	Improved internet bandwidth		
12	Training through existing recognized platforms		
13	Multisectoral and integrated training		
14	Short training session time		
15	Protected work time for training		

Supplementary Table 2: First intercoder reliability (percent agreement statistics)

Percent agreement for MOT constraints and enablers between first coder and second coder[2]

Key:
0= Agreement (i.e., both coders agree for either MOT constraint or enabler)
1= Disagreement (i.e., both coders disagree for either MOT constraint or enabler)

Constraint construct	Coder 1	Coder 2	Differences
Constraint 1 construct	AR	AR	0
Constraint 2 construct	AR	AR	0
Constraint 3 construct	CS	CS	0
Constraint 4 construct	DE	DE	0
Constraint 5 construct	DE	DE	0
Constraint 6 construct	CA	CA	0
Constraint 7 construct	CO	AD	1
Constraint 8 construct	SC	SC	0
Constraint 9 construct	LC	LC	0
Constraint 10 construct	PL	PL	0
Constraint 11 construct	PL	PL	0
Constraint 12 construct	EN	TE	1
Constraint 13 construct	PA	PA	0
Constraint 14 construct	AC	PA	1
Constraint 15 construct	COM	COM	0
Number of zeros			*12*
Number of constructs			*15*
Percent agreement			***80.0%***
**Enabler construct**			
Enabler 1 construct	AR	AR	0
Enabler 2 construct	AA	AD	1
Enabler 3 construct	AD	AD	0
Enabler 4 construct	CA	CA	0
Enabler 5 construct	PL	PL	0
Enabler 6 construct	EN	EN	0
Enabler 7 construct	AKI	AKI	0
Enabler 8 construct	RC	RC	0
Enabler 9 construct	AC	PL	1
Enabler 10 construct	LC	LC	0
Enabler 11 construct	LC	LC	0
Enabler 12 construct	DE	DE	0
Enabler 13 construct	DE	DE	0
Enabler 14 construct	DE	DE	0
Enabler 15 construct	PL	PL	0
Number of zeros			*13*
Number of constructs			*15*
Percent agreement			***86.7%***
**Constraint and enabler construct**			
Number of zeros			*25*
Number of constructs			*30*
Percent agreement			***83.3%***

Supplementary Table 3: Second intercoder reliability (percent agreement statistics)

Percent agreement for MOT constraints and enablers between initial coding and final coding[2]

Key:
0= Agreement (i.e., both codings agree for either MOT constraint or enabler)
1= Disagreement (i.e., both codings disagree for either MOT constraint or enabler)

Constraint construct	Initial coding	Final coding	Differences
Constraint 1 construct	AR	AR	0
Constraint 2 construct	AR	AR	0
Constraint 3 construct	CS	CS	0
Constraint 4 construct	DE	DE	0
Constraint 5 construct	DE	DE	0
Constraint 6 construct	CA	CA	0
Constraint 7 construct	CO	CO	0
Constraint 8 construct	SC	SC	0
Constraint 9 construct	LC	LC	0
Constraint 10 construct	PL	PL	0
Constraint 11 construct	PL	PL	0
Constraint 12 construct	TE	EN	1
Constraint 13 construct	PA	PA	0
Constraint 14 construct	AC	AN	1
Constraint 15 construct	COM	COM	0
Number of zeros			*13*
Number of constructs			*15*
Percent agreement			***86.7%***
**Enabler construct**			
Enabler 1 construct	AR	AR	0
Enabler 2 construct	AD	AA	1
Enabler 3 construct	AD	AD	0
Enabler 4 construct	CA	CA	0
Enabler 5 construct	PL	PL	0
Enabler 6 construct	EN	EN	0
Enabler 7 construct	AKI	AKI	0
Enabler 8 construct	RC	RC	0
Enabler 9 construct	AC	AN	1
Enabler 10 construct	LC	LC	0
Enabler 11 construct	LC	LC	0
Enabler 12 construct	DE	DE	0
Enabler 13 construct	DE	DE	0
Enabler 14 construct	DE	DE	0
Enabler 15 construct	PL	PL	0
Number of zeros			*13*
Number of constructs			*15*
Percent agreement			***86.7%***
**Constraint and enabler construct**			
Number of zeros			*26*
Number of constructs			*30*
Percent agreement			***86.7%***

Supplementary Figures 1-3: First intercoder reliability (Cohen's kappa statistics)

Cohen's kappa calculation for MOT constraints and enablers between first coder and second coder[2]

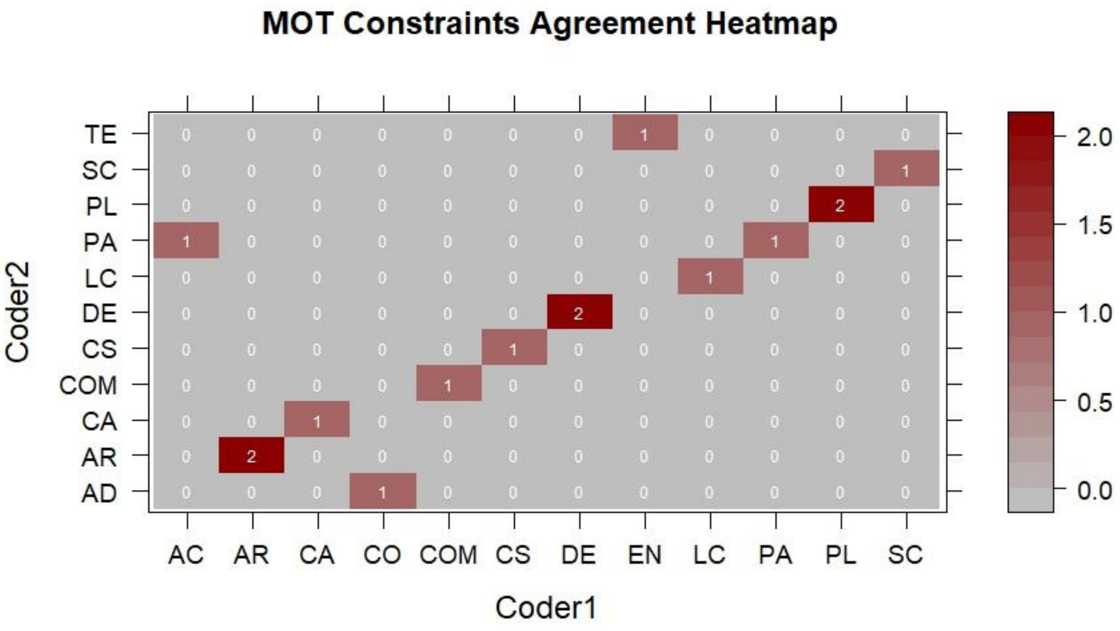

Figure 1. A confusion matrix heatmap for multi-communication online training (MOT) constraints coding agreement between coder 1 and coder 2.

1a. Statistical Estimation of Kappa for MOT Constraints Individual Coding

Cohen's Kappa for 2 Raters (Weights: unweighted) using the *irr* package in R.

Subjects = 15
Raters = 2
Kappa (κ) = 0.7815534 = **0.78**
z = 10.3
p-value = 0

1b. Mathematical Derivation of Kappa for MOT Constraints Individual Coding

Observed percent agreement (Po) = 0.800

Chance percent agreement (Pe) = $\frac{c1 \times r1 + c2 \times r2 + c3 \times r3 + \ldots c11 \times r11}{N^2}$

Where, $c1$ is column 1 marginal sum and $r1$ is row 1 marginal sum.

$$= \frac{1\times1 + 2\times2 + 1\times1 + 1\times1 + 1\times1 + 1\times2 + 2\times1 + 1\times2 + 1\times2 + 1\times1 + 2\times1}{15^2}$$

$$= \frac{1 + 4 + 1 + 1 + 1 + 2 + 2 + 2 + 2 + 1 + 2}{225}$$

$$= \frac{19}{225} = 0.084$$

$$\kappa = \frac{Po - Pe}{1 - Pe}$$

$$= \frac{0.800 - 0.084}{1 - 0.084}$$

$$= \frac{0.716}{0.916} = \mathbf{0.78}$$

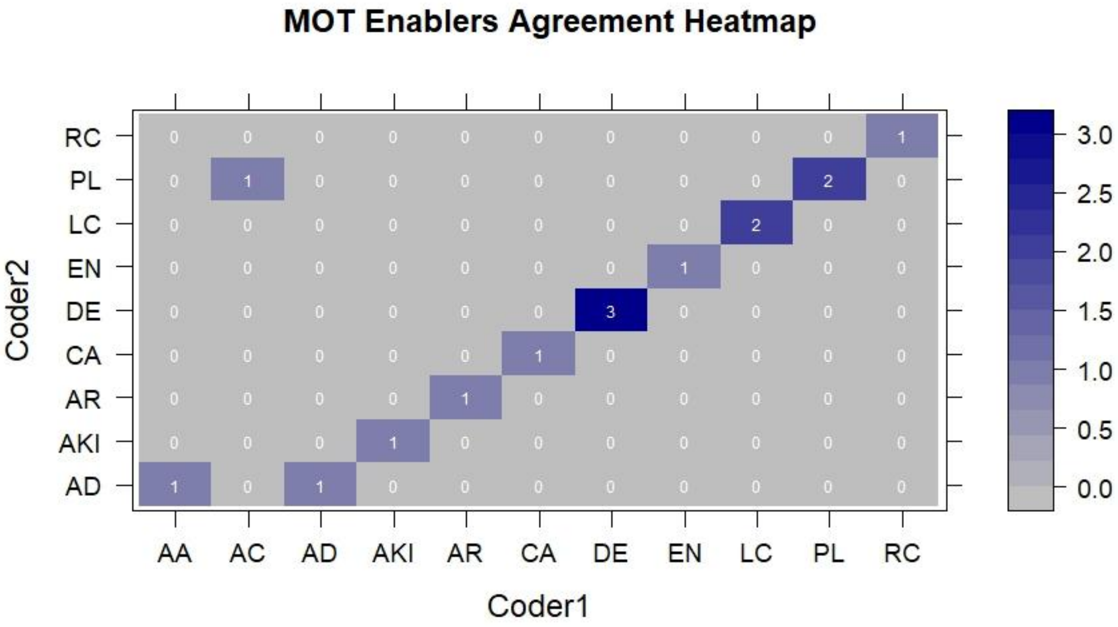

Figure 2. A confusion matrix heatmap for multi-communication online training (MOT) enablers coding agreement between coder 1 and coder 2.

2a. Statistical Estimation of Kappa for MOT Enablers Individual Coding
Cohen's Kappa for 2 Raters (Weights: unweighted) using the *irr* package in R.

Subjects = 15
Raters = 2
Kappa (κ) = 0.8492462 = **0.85**
z = 9.46
p-value = 0

2b. Mathematical Derivation of Kappa for MOT Enablers Individual Coding
Observed percent agreement (Po) = 0.867

Chance percent agreement (Pe) = $\frac{c1 \times r1 + c2 \times r2 + c3 \times r3 + \dots c9 \times r9}{N^2}$

Where, $c1$ is column 1 marginal sum and $r1$ is row 1 marginal sum.

$$= \frac{1\times2 + 1\times1 + 1\times1 + 1\times1 + 1\times3 + 1\times1 + 3\times2 + 1\times3 + 2\times1}{15^2}$$

$$= \frac{2 + 1 + 1 + 1 + 3 + 1 + 6 + 3 + 2}{225}$$

$$= \frac{20}{225} = 0.089$$

$$\kappa = \frac{Po - Pe}{1 - Pe}$$

$$= \frac{0.867 - 0.089}{1 - 0.089}$$

$$= \frac{0.778}{0.911} = \mathbf{0.85}$$

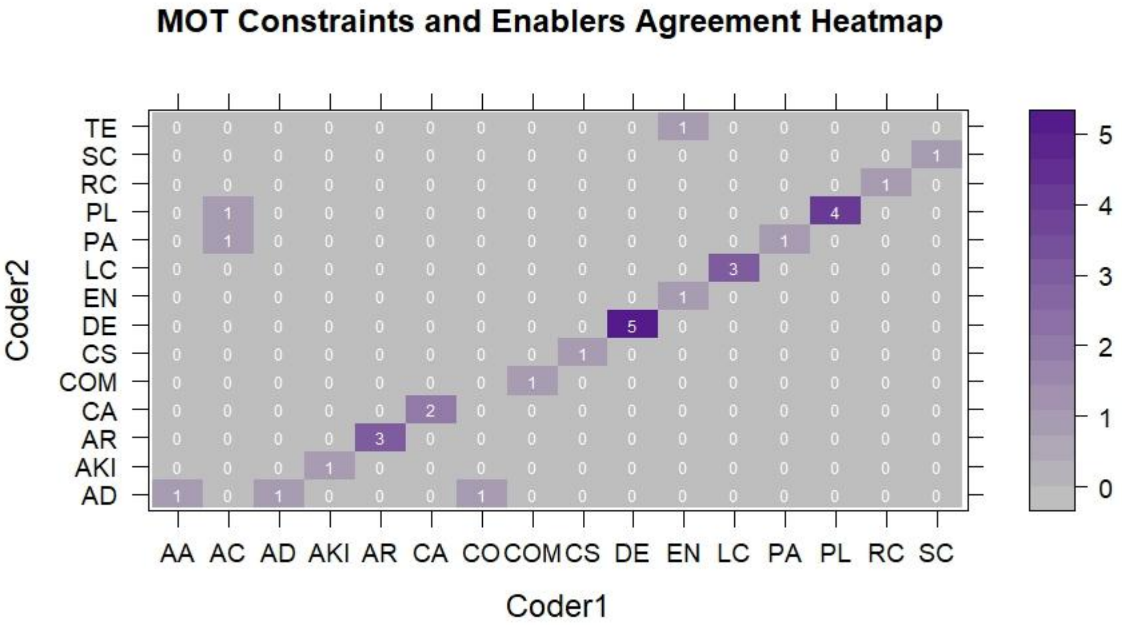

Figure 3. A confusion matrix heatmap for multi-communication online training (MOT) constraints and enablers coding agreement between coder 1 and coder 2.

3a. Statistical Estimation of Kappa for MOT Constraints and Enablers Individual Coding
Cohen's Kappa for 2 Raters (Weights: unweighted) using the *irr* package in R.

Subjects = 30
Raters = 2
Kappa (κ) = 0.817296 = **0.82**
z = 15
p-value = 0

3b. Mathematical Derivation of Kappa for MOT Constraints and Enablers Individual Coding
Observed percent agreement (Po) = 0.833

Chance percent agreement (Pe) = $\frac{c1 \times r1 + c2 \times r2 + c3 \ldots c14 \times r14}{N^2}$

Where, $c1$ is column 1 marginal sum and $r1$ is row 1 marginal sum.

$$= \frac{1\times3 + 2\times1 + 1\times3 + 1\times2 + 3\times2 + 2\times1 + 1\times5 + 1\times1 + 1\times3 + 5\times2 + 2\times5 + 3\times1 + 1\times1 + 4\times1}{30^2}$$

$$= \frac{3 + 2 + 3 + 2 + 6 + 2 + 5 + 1 + 3 + 10 + 10 + 3 + 1 + 4}{900}$$

$$= \frac{55}{900} = 0.061$$

$$\kappa = \frac{Po - Pe}{1 - Pe}$$

$$= \frac{0.833 - 0.061}{1 - 0.061}$$

$$= \frac{0.772}{0.939} = \mathbf{0.82}$$

Supplementary Figures 4-6: Second intercoder reliability (Cohen's kappa statistics)

Cohen's kappa calculation for MOT constraints and enablers between initial coding and final coding[2]

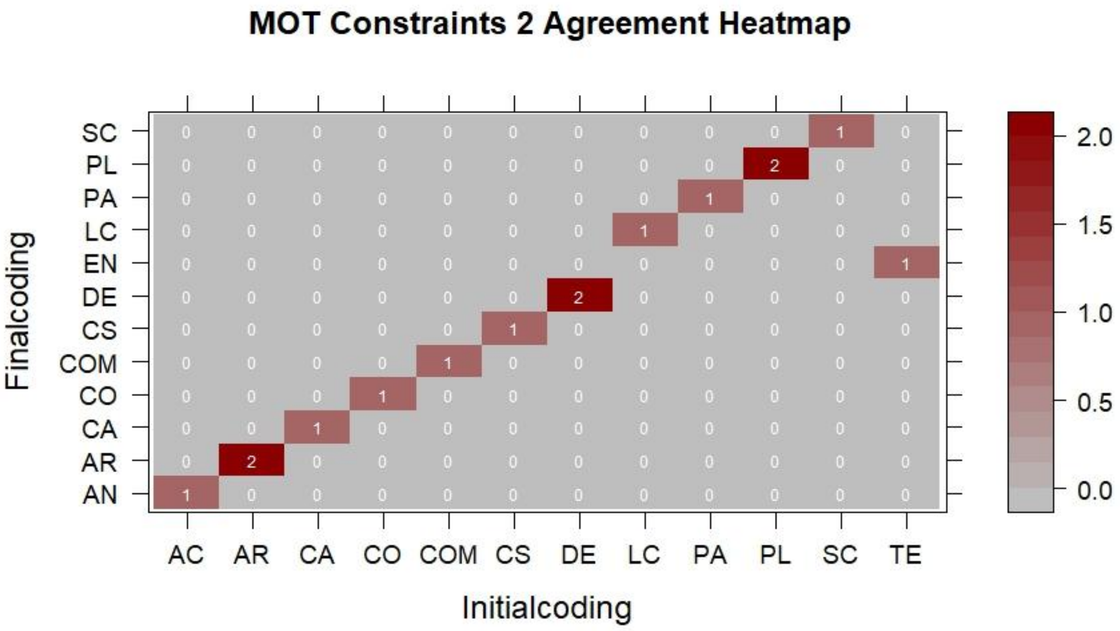

Figure 4. A confusion matrix heatmap for multi-communication online training (MOT) constraints coding agreement between initial coding and final coding.

4a. Statistical Estimation of Kappa for MOT Constraints Team Coding
Cohen's Kappa for 2 Raters (Weights: unweighted) using the *irr* package in R.

Subjects = 15
Raters = 2
Kappa (κ) = 0.8543689 = **0.85**
z = 11.2
p-value = 0

4b. Mathematical Derivation of Kappa for MOT Constraints Team Coding
Observed percent agreement (Po) = 0.867

Chance percent agreement (Pe) = $\frac{c1 \times r1 + c2 \times r2 + c3 + \ldots c12 \times r12}{N^2}$

Where, $c1$ is column 1 marginal sum and $r1$ is row 1 marginal sum.

$= \frac{1\times1 + 2\times2 + 1\times1 + 1\times1 + 1\times1 + 1\times1 + 2\times2 + 1\times1 + 1\times1 + 2\times1 + 1\times2 + 1\times1}{15^2}$

$= \frac{1 + 4 + 1 + 1 + 1 + 1 + 4 + 1 + 1 + 2 + 2 + 1}{225}$

$= \frac{18}{225} = 0.080$

$$\kappa = \frac{Po - Pe}{1 - Pe}$$

$$= \frac{0.867 - 0.080}{1 - 0.080}$$

$$= \frac{0.787}{0.920} = \mathbf{0.85}$$

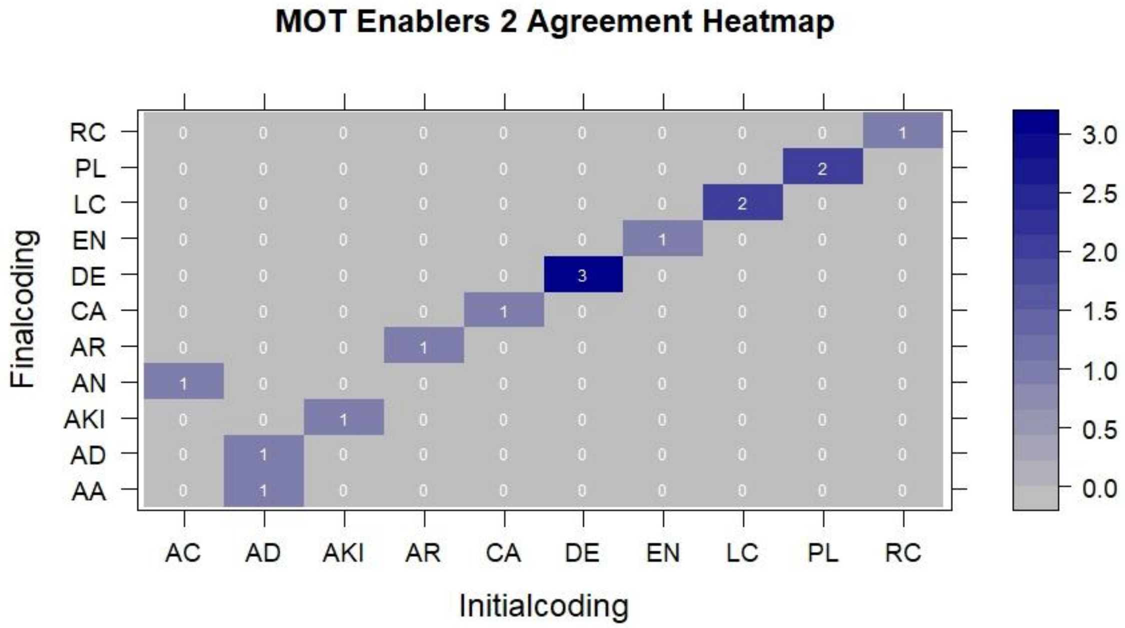

Figure 5. A confusion matrix heatmap for multi-communication online training (MOT) enablers coding agreement between initial coding and final coding.

5a. Statistical Estimation of Kappa for MOT Enablers Team Coding
Cohen's Kappa for 2 Raters (Weights: unweighted) using the *irr* package in R.

Subjects = 15
Raters = 2
Kappa (κ) = 0.8507463 = **0.85**
z = 9.93
p-value = 0

5b. Mathematical Derivation of Kappa for MOT Enablers Team Coding
Observed percent agreement (Po) = 0.867

Chance percent agreement (Pe) = $\dfrac{c1 \times r1 + c2 \times r2 + c3 + \ldots c10 \times r10}{N^2}$

Where, $c1$ is column 1 marginal sum and $r1$ is row 1 marginal sum.

$$= \frac{1\times1 + 2\times1 + 1\times1 + 1\times1 + 1\times1 + 3\times1 + 1\times3 + 2\times1 + 2\times2 + 1\times2}{15^2}$$

$$= \frac{1+2+1+1+1+3+3+2+4+2}{225}$$

$$= \frac{20}{225} = 0.089$$

$$\kappa = \frac{Po - Pe}{1 - Pe}$$

$$= \frac{0.867 - 0.089}{1 - 0.089}$$

$$= \frac{0.778}{0.911} = \mathbf{0.85}$$

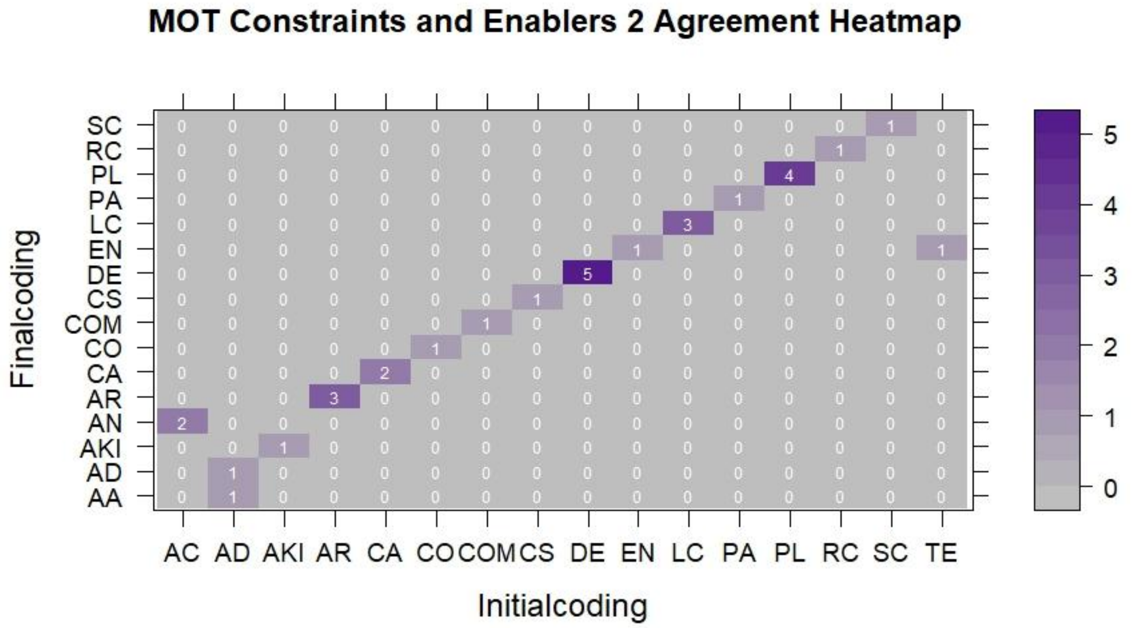

Figure 6. A confusion matrix heatmap for multi-communication online training (MOT) constraints and enablers coding agreement between initial coding and final coding.

6a. Statistical Estimation of Kappa for MOT Constraints and Enablers Team Coding
Cohen's Kappa for 2 Raters (Weights: unweighted) using the *irr* package in R.

Subjects = 30
Raters = 2
Kappa (κ) = 0.8547215 = **0.85**
z = 16.3
p-value = 0

6b. Mathematical Derivation of Kappa for MOT Constraints and Enablers Team Coding
Observed percent agreement (Po) = 0.867

Chance percent agreement (Pe) = $\frac{c1 \times r1 + c2 \times r2 + c3 + \ldots c16 \times r16}{N^2}$

Where, $c1$ is column 1 marginal sum and $r1$ is row 1 marginal sum.

$$= \frac{2\times1 + 2\times1 + 1\times1 + 3\times2 + 2\times3 + 1\times2 + 1\times1 + 1\times1 + 5\times1 + 1\times5 + 3\times2 + 1\times3 + 4\times1 + 1\times4 + 1\times1 + 1\times1}{30^2}$$

$$= \frac{2 + 2 + 1 + 6 + 6 + 2 + 1 + 1 + 5 + 5 + 6 + 3 + 4 + 4 + 1 + 1}{900}$$

$$= \frac{50}{900} = 0.056$$

$$\kappa = \frac{Po - Pe}{1 - Pe}$$

$$= \frac{0.867 - 0.056}{1 - 0.056}$$

$$= \frac{0.811}{0.944} = \mathbf{0.85}$$

Supplementary Discussion: Future directions

Regarding application of CFIR prospectively and as a guide, one of the ways the confirmed association or relationship of implementation determinants with implementation context can be assessed is by conducting inferential analytics using statistical methods such as multivariable logistic or log-binomial regression or and Cramer's V or tetrachoric correlation, respectively. This is necessary to account for the confounding effects of other implementation determinants in CFIR or those synthesized from the literature or expert consensus. This would help in evaluating the findings: 1) validity, with statistical significance or correlation relative to their clinical or real-world significance at the overall study population unit of analysis, and 2) reliability, with subgroup analysis at the work discipline, health sectors, work area, and country income group units of analysis, to inform accurate and targeted design of implementation strategies. Concerning application of CFIR as a tool development and validation, these can be determined through psychometric analysis such as content validity index or confirmatory factor analysis and Cohen's kappa or intraclass correlation, respectively. Similarly, this would help in evaluating the validity and reliability of findings at the overall study population and subgroup units of analysis, to inform accurate and universal utility of the tool. The effects of e-learning ICD on implementation success compared to the traditional face-to-face (F2F) modality can also be assessed to improve our understanding of their impact beyond significance. Like in a randomized controlled trial study, these estimates can be approximated in a cross-sectional study with the same participants completing responses for both e-learning and F2F modalities. This is possible given no changes in the baseline characteristics of participants and the possible restriction of analysis to those with a "Yes" response to CFIR-derived implementation contexts and determinants with or without statistically significant associations or moderate-strong relationships.

References

1. Damschroder, LJ. et al. The updated Consolidated Framework for Implementation Research based on user feedback. *Implement Sci*. **17**, 75 (2022).

2. McHugh ML. Interrater reliability: the kappa statistic. *Biochem Med (Zagreb)*. **22**(3), 276–82 (2012).
